# Development and Validation of a Burkholderia pseudomallei Core Genome Multilocus Sequence Typing Scheme To Facilitate Molecular Surveillance

**DOI:** 10.1128/JCM.00093-21

**Published:** 2021-07-19

**Authors:** Sabine Lichtenegger, Trung T. Trinh, Karoline Assig, Karola Prior, Dag Harmsen, Julian Pesl, Andrea Zauner, Michaela Lipp, Tram A. Que, Beatrice Mutsam, Barbara Kleinhappl, Ivo Steinmetz, Gabriel E. Wagner

**Affiliations:** a Institute of Hygiene, Microbiology and Environmental Medicine, Medical University of Graz, Graz, Austria; b VNU-Institute of Microbiology and Biotechnology, Vietnam National University, Hanoi, Vietnam; c Department of Periodontology and Operative Dentistry, University Hospital Münster, Münster, Germany; d General Hospital of Nghe An Province, Nghe An, Vietnam; University of Iowa College of Medicine

**Keywords:** *Burkholderia pseudomallei*, WGS-based typing method, core genome multilocus sequence typing, melioidosis, molecular surveillance

## Abstract

Burkholderia pseudomallei causes the severe disease melioidosis. Whole-genome sequencing (WGS)-based typing methods currently offer the highest resolution for molecular investigations of this genetically diverse pathogen. Still, its routine application in diagnostic laboratories is limited by the need for high computing power, bioinformatic skills, and variable bioinformatic approaches, with the latter affecting the results. We therefore aimed to establish and validate a WGS-based core genome multilocus sequence typing (cgMLST) scheme, applicable in routine diagnostic settings. A soft defined core genome was obtained by challenging the B. pseudomallei reference genome K96243 with 469 environmental and clinical genomes, resulting in 4,221 core and 1,351 accessory targets. The scheme was validated with 320 WGS data sets. We compared our novel typing scheme with single nucleotide polymorphism-based approaches investigating closely and distantly related strains. Finally, we applied our scheme for tracking the environmental source of a recent infection. The validation of the scheme detected >95% good cgMLST target genes in 98.4% of the genomes. Comparison with existing typing methods revealed very good concordance. Our scheme proved to be applicable to investigating not only closely related strains but also the global B. pseudomallei population structure. We successfully utilized our scheme to identify a sugarcane field as the presumable source of a recent melioidosis case. In summary, we developed a robust cgMLST scheme that integrates high resolution, maximized standardization, and fast analysis for the nonbioinformatician. Our typing scheme has the potential to serve as a routinely applicable classification system in B. pseudomallei molecular epidemiology.

## INTRODUCTION

The environmental bacterium Burkholderia pseudomallei causes melioidosis, which presents a wide clinical spectrum ranging from localized infections to multiple-organ involvement and the most severe outcome, sepsis. Global modeling predicted 165,000 human melioidosis cases annually, with 89,000 deaths (1), calling for substantial improvements in surveillance and laboratory resources to verify these predictions. The rate of mortality caused by melioidosis varies from 10% in northern Australia to 40% in Thailand and other locations where the disease is endemic, highlighting the need for global surveillance programs.

B. pseudomallei molecular surveillance is essential for (i) identifying sources of infection, (ii) characterizing the nature and mechanism of global transmission, and (iii) testing the hypothesis that some genetic profiles are more frequently associated with certain clinical outcomes.

The reliability of methods for molecular surveillance strongly depends on the discriminatory power of the typing technique. Whole-genome sequencing (WGS)-based typing outcompetes multilocus sequence typing (MLST) and pulsed-field gel electrophoresis (PFGE) due to its markedly increased resolution (2) and high interlaboratory reproducibility (3). WGS-based single nucleotide polymorphism (SNP) calling offers the highest currently available resolution and is well established for B. pseudomallei (4–7). However, prerequisites for these analyses are access to high computing power, the choice of a representative reference genome, and an array of variable bioinformatics tools, with the latter two potentially influencing the outcome.

We therefore aimed to develop and validate a standardized and universally applicable core genome MLST (cgMLST) scheme for B. pseudomallei with high discriminatory power and modest computational demands that is applicable for closely as well as distantly related isolates (4). Similar to conventional MLST, it is based on gene-by-gene allelic profiling but exploits thousands of genes across the entire genome. Thereby, it combines an easily expandable and standardized classification scheme with the resolution of a WGS-based method (8, 9). It further offers the capacity to analyze dozens of genomes on a standard computer in a reasonable amount of time and simple expansion of an existing collection of strains without retyping all isolates.

We demonstrate the efficacy, high resolution, and concordance of B. pseudomallei cgMLST by comparing it to published SNP analyses of globally distributed clinical and environmental strains. Moreover, we performed cgMLST on a melioidosis case in Vietnam and epidemiologically linked soil isolates and thereby localized the suspected source of infection.

## MATERIALS AND METHODS

### DNA extraction, WGS, and assembly.

DNA was extracted as previously described (10). K96243 libraries were prepared using the Nextera XT library preparation kit (Illumina, Inc., San Diego, CA, USA) according to the manufacturer’s recommendations. Next-generation sequencing (NGS) was performed on an Illumina MiSeq instrument using MiSeq reagent kit version 2 (Illumina) for a 2- by 250-bp paired-end sequencing run. For all other isolates, NGS was performed on an Illumina HiSeq instrument using HiSeq SBS kit version 4 (Illumina) for a 2- by 100-bp paired-end sequencing run. Raw data were assembled with SKESA (default setting, version 2.3.0) (11) implemented in SeqSphere^+^ (version 6.0.92; Ridom GmbH, Münster, Germany).

### Data acquisition for a soft defined cgMLST scheme.

A global, diverse B. pseudomallei collection (5) was used for the definition of the core genome MLST scheme. FASTA files of 92 strains were downloaded from the NCBI genome database, and FASTQ files from 377 strains were downloaded from the NCBI Sequence Read Archive (SRA). SRA data sets were assembled with SKESA with default settings, implemented in SeqSphere^+^.

### Preliminary target scheme definition.

The finished, publicly available genome of B. pseudomallei K96243 listed as a representative genome on the NCBI genome homepage with a genome status of complete (version 1; GenBank accession no. NC_006350.1 [11 January 2017] and NC_006351.1 [3 August 2016]) was selected as the seed genome for cgMLST scheme definition. Using the cgMLST Target Definer tool (version 1.5 with default parameters) (SeqSphere^+^; Ridom), a rapid local *ad hoc* cgMLST scheme was defined that contained all genes of the seed genome that were not homologous, did not contain internal stop codons, and did not overlap other genes. Genes that matched B. pseudomallei plasmid sequences (3 NCBI entries, 3 June 2019) were excluded during scheme definition. A total of 468 data sets of the collection of Chewapreecha et al. (5) were chosen for soft cgMLST definition and were analyzed with the preliminary reference task template. Targets from the *ad hoc* scheme present within <97% of the data sets were manually deleted from the preliminary cgMLST scheme and moved to the accessory task template.

### Optimization of the cgMLST scheme for draft genomes.

The assembly of fragments with short read lengths usually results in draft genomes with several contiguous sequences lacking parts of the genome due to the many repetitive genetic elements in bacteria (12). Since bacterial genomics is currently dominated by short-read sequencing platforms, it is important to identify regions in the B. pseudomallei genome that are lost more often. Therefore, genomic DNA of the seed genome strain K96243 was isolated and sequenced in triplicates. Raw data were assembled with SKESA (default settings, as implemented in SeqSphere^+^), and assembled draft genomes were analyzed with the preliminary cgMLST scheme. Targets of the scheme missing in more than one of the three data sets were regarded as potentially problematic, manually excluded from the preliminary cgMLST scheme, and transferred to the accessory task template. The remaining targets (see Table S1 in the supplemental material) were defined as core genome genes and used for the subsequent typing scheme.

### Phylogenetic trees.

The allelic profile, i.e., the combination of alleles of the found target genes per sample, was used to generate minimum-spanning trees (MSTs) or neighbor-joining (NJ) trees by mutual comparison of each allele of the found target genes and summing up the number of different alleles between two isolates where possible (missing target genes were excluded by choosing the parameter “pairwise ignore missing values”). Figures were created and annotated with Ridom SeqSphere^+^. For the SNP-based NJ tree shown in Fig. S1 in the supplemental material, core genome SNPs were extracted from Ridom SeqSphere^+^, and the tree was generated using molecular evolutionary genetics analysis (MEGA).

### Isolates and culture conditions.

Environmental B. pseudomallei strains were isolated as previously described (13). For bacterial DNA isolation, the strains were cultivated on Columbia agar supplemented with 5% sheep blood overnight. Strain information and the accession numbers for the corresponding sequencing data are listed in Table 1.

**TABLE 1:** B. pseudomallei strains isolated and sequenced in this study

Isolate	Sample type	Yr of isolation	NCBI accession no.
AH04	Soil	2017	ERR5011042
AH09	Soil	2017	ERR5011043
AH16	Soil	2017	ERR5011045
AH21	Soil	2017	ERR5011047
AH26	Soil	2017	ERR5011048
AH32	Soil	2017	ERR5011049
AH33	Soil	2017	ERR5011050
AH34	Soil	2017	ERR5011046
AH36	Soil	2017	ERR5011044
NA76	Blood	2017	ERR5011051
NA77	Pus	2017	ERR5011052
NA18	Blood	2018	ERR5011053

### Data availability.

Raw reads were submitted to the European Nucleotide Archive (http://www.ebi.ac.uk/ena/) under the study accession number PRJEB42163.

## RESULTS

### High-resolution typing scheme for B. pseudomallei.

To ensure a typing scheme suitable for all strains of a species, it is important to consider the genetic variability when creating a core genome. Therefore, we applied a common approach that uses a large number of genomes to infer a “soft defined core genome” (14). The scheme is established by defining a specific percentage threshold, e.g., the presence of all core genes/targets in >95% of analyzed genomes (15). Chewapreecha et al. (5) reported a genome collection consisting of environmental and clinical B. pseudomallei isolates collected in 30 countries over a period of 79 years (5). A total of 468 genomes from this collection were utilized to maximize the distribution over time and geography, with representatives from each continent meeting all the requirements for generating a stable cgMLST scheme. We carried out a conventional MLST analysis, which revealed 211 different sequence types (STs) present in the collection, again indicating great diversity.

A preliminary reference task template was generated using the K96243 reference genome, resulting in 5,580 target genes after thorough filtering. Using the 468 genome sequences, a cgMLST scheme of 4,221 core and 1,351 accessory genome targets was defined. The cgMLST scheme was challenged with 320 genomes in a literature data-based validation. In 98.4% of theses genomes, >95% good cgMLST targets were detected (on average, 99.4%).

### Validation of the cgMLST scheme by comparison to existing methods.

**FIG 1:**
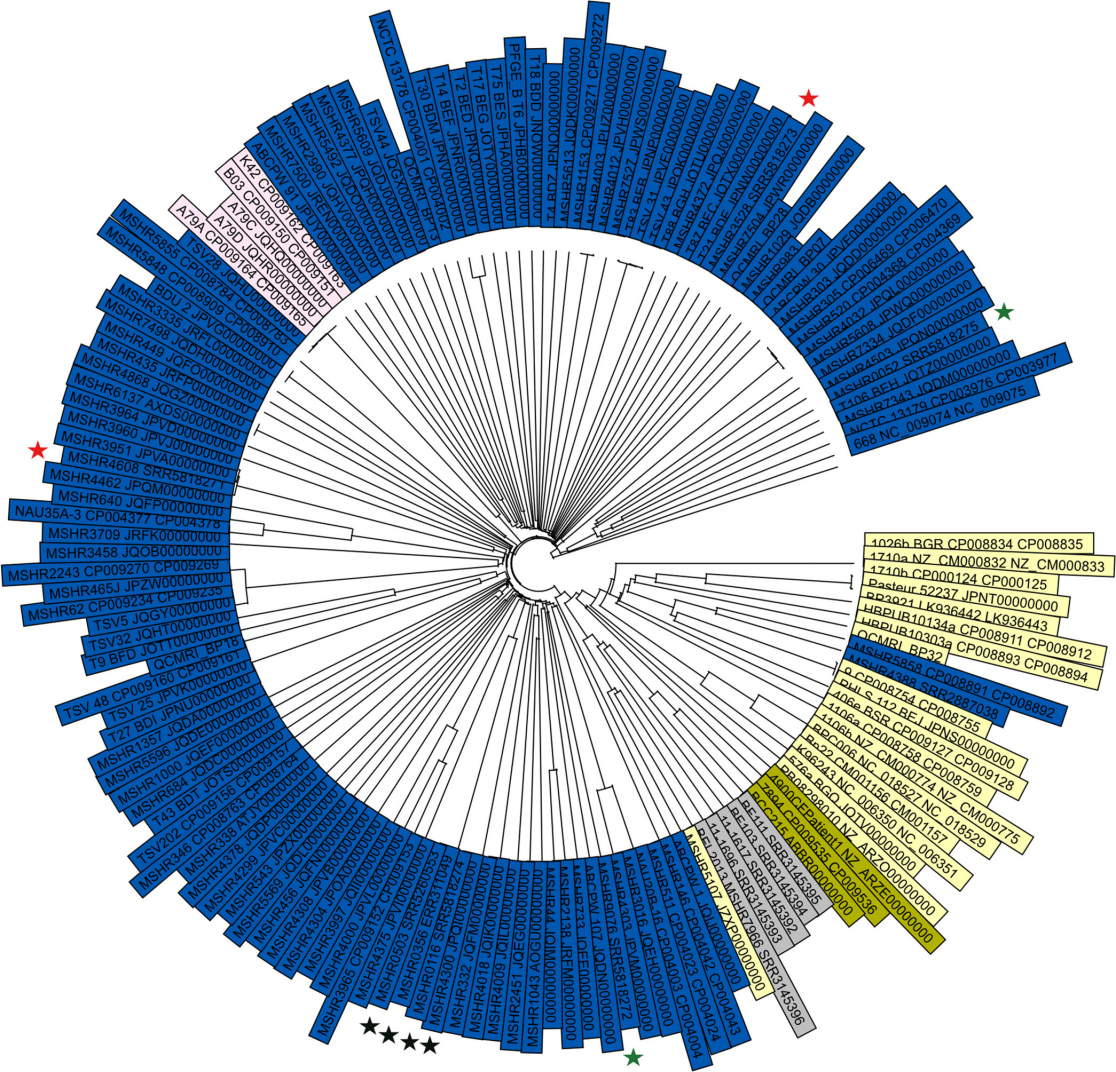
cgMLST UPGMA tree of 150 global B. pseudomallei isolates constructed using 4,221 target genes. K96243 was used as the reference strain. Colors represent the origin of strains (blue, Australia; red, Papua New Guinea; yellow, Asia; gray, Africa; green, South America). Isolates that were subjected to homoplasy analysis are marked by stars. Black stars mark true long-range dispersal, while red and green stars mark homoplasy events.

**(i) Global phylogenetic analysis.** Analysis of a collection of global B. pseudomallei isolates (16) demonstrated the high resolution power of cgMLST. The 150 isolates were resolved into 148 types (Simpson’s diversity index, 1.00 [95% confidence interval {CI}, 0.99 to 1.00]). The cgMLST-based UPGMA tree (Fig. 1) shows clustering of global isolates similar to that of a previously constructed global SNP phylogeny (16) and provides high resolution for closely related isolates on a global level. Moreover, in accordance with SNP-based variant calling, cgMLST identified two previously described intracontinental homoplasy events (Fig. 1, red and green stars) and a true long-range dispersal of an identical MLST ST (Fig. 1, black stars) (6).

**FIG 2:**
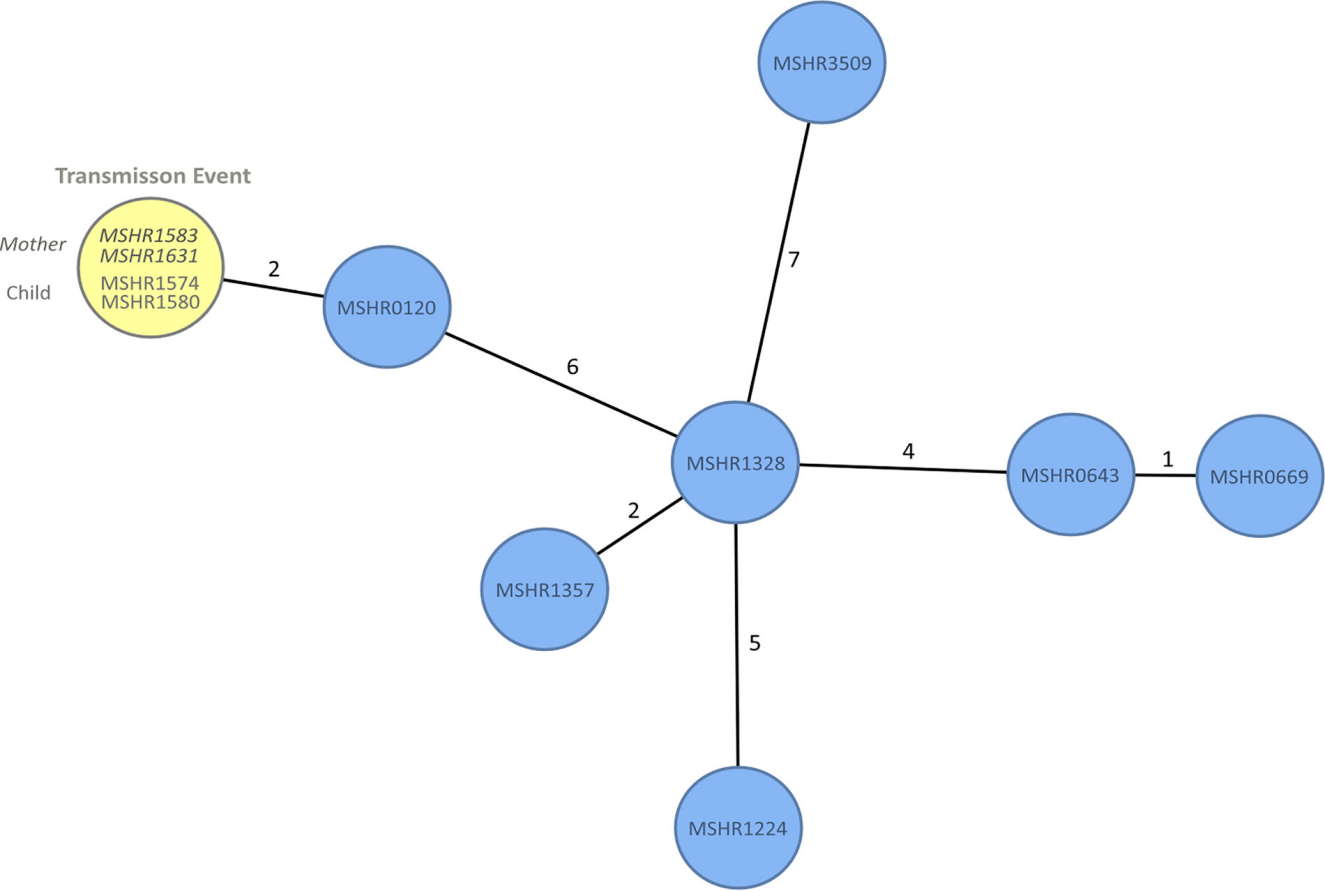
cgMLST minimum-spanning tree, including isolates from a previously described human-to-human transmission event (4). Four isolates related to the transmission event (yellow) and unrelated clinical isolates sharing the same MLST ST (blue) are depicted. Each circle represents an allelic profile based on sequence analysis of 4,221 target genes. The numbers on the connecting lines refer to the number of allele differences.

**(ii) Reinvestigation of a human-to-human transmission event by cgMLST.** We reanalyzed a B. pseudomallei human-to-human transmission event (4), as these events represent a severe challenge for the resolution power of a typing method. The data set included isolates from an infected mother and the infected child and other clinical isolates sharing the same MLST ST. cgMLST revealed identical allelic profiles for all mother and child isolates (Fig. 2, yellow). This is in agreement with SNP calling, where no difference between these isolates was observed (4). The above-mentioned study also described a clinical isolate from 1992 (MSHR0120), which differs in only seven SNP-indel variants compared to the strains from the transmission event. Even such a small genomic mismatch could be resolved by cgMLST (two-allele difference) (Fig. 2). The resolution power of our novel typing scheme was further corroborated by the analysis of MSHR1357 and MSHR1328 for which SNP analysis revealed solely five variants (4) corresponding to two alleles in our cgMLST tree (Fig. 2). cgMLST resolved all tested strains sharing identical MLST STs and performed comparably well as SNP-indel analysis.

**FIG 3:**
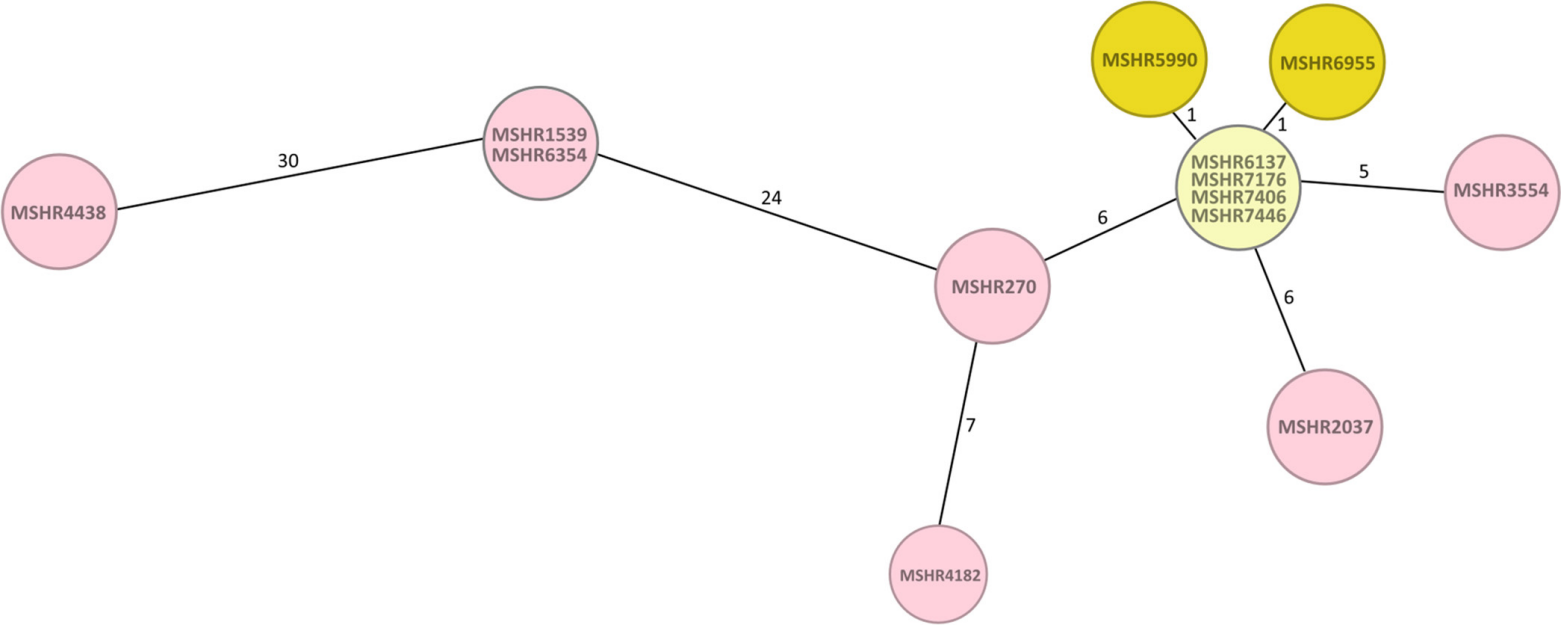
cgMLST minimum-spanning tree, including environmental (light yellow) and clinical (dark yellow) isolates from a previously described melioidosis outbreak in northern Australia and isolates collected within a 10-km radius (red) (7). Each circle represents an allelic profile based on sequence analysis of 4,221 target genes. The numbers on the connecting lines refer to the number of allele differences.

**(iii) Outbreak source tracing.** The precise link to the environmental source of an infection often remains obscure. MLST is a good predictor of the exposure site but is sometimes insufficient to pinpoint it down to a concrete location. We reanalyzed patient and environmental isolates, which were indistinguishable on the conventional MLST level but represent distinct strains as revealed by SNP-indel analysis (7). cgMLST analysis confirmed a domestic water supply as the source of infection for two melioidosis cases from the same residential property. The two clinical isolates (MSHR5990 and MSHR6955) (Fig. 3, dark yellow) differed from the water supply isolate (MSHR6137) (light yellow) by one allele and from each other by two alleles (Fig. 3), which corresponds to 6 SNPs and 12 SNPs identified previously (7). cgMLST profiling did not resolve the environmental isolates (MSHR6137, MSHR7176, MSHR7406, and MSHR7446) (Fig. 3, light yellow) sampled at the location epidemiologically associated with the two patients (MSHR5990 and MSHR6955) (dark yellow). Noteworthy, these isolates differed in just a few SNPs, demonstrating their close phylogenetic relationship. cgMLST additionally linked the patient isolate MSHR6354 to the environmental strain MSHR1539 but did not resolve these two isolates, which were distinguishable by SNP-indel analysis. Nevertheless, cgMLST resolution was by far sufficient to reconfirm the previously identified source of infection.

### (iv) cgMLST analysis of a recent melioidosis case reveals the environmental exposure site.

**FIG 4:**
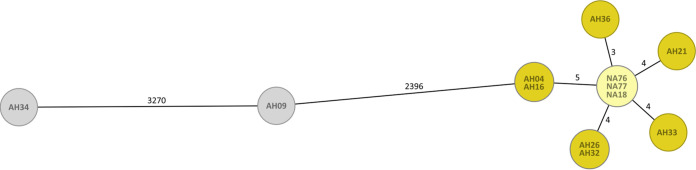
cgMLST minimum-spanning tree, including clinical (light yellow) isolates from a melioidosis outbreak in Vietnam and environmental isolates (dark yellow or gray) collected at the potential infection site. Each circle represents an allelic profile based on sequence analysis of 4,221 target genes. The numbers on the connecting lines refer to the number of allele differences.

We applied our validated scheme for tracing the environmental source of a recent human B. pseudomallei infection in Vietnam. In July 2017, a culture-proven melioidosis case was reported in Chau Dinh commune, Quy Hop district, Nghe An province. We sequenced two strains (from blood and pus) and a relapse isolate from the same patient in November 2018. All three isolates (NA76 [blood], NA77 [pus], and NA18 [blood; relapse]) (Fig. 4, light yellow) shared identical allelic profiles. To investigate the environmental source of the infection, we carried out sampling at the potential infection site, a sugarcane field in the proximity of the patient’s home. We sequenced nine environmental B. pseudomallei isolates (each environmental isolate starts with the prefix “AH” followed by a number). We found two environmental B. pseudomallei isolates that differed from all other strains in thousands of alleles. Within the patient cluster, we observed environmental isolates that differed in only 3 to 5 alleles from the clinical strains, categorizing the sugarcane field as the presumed exposure site. An NJ tree based on cgMLST SNPs shows high concordance with the cgMLST allele-based tree (see Fig. S1 in the supplemental material).

## DISCUSSION

With the continuous decrease in costs and its constant methodological improvement, WGS has become available for routine diagnostic applications. Therefore, it is critical to develop rapid and standardized WGS-based typing methods. A number of recent studies demonstrate that WGS-based cgMLST of bacterial pathogens (3, 17–21) combines superior resolution with rapid, simple, and standardized analysis.

In this study, we demonstrate that our B. pseudomallei cgMLST scheme has the discriminatory power to identify potential transmission events (Fig. 2). Our analyses showed that it was possible to resolve closely related strains, which differ in no more than five SNPs, corresponding to two allele differences in the example provided here. Noteworthy, a certain number of SNP-indel mutations does not translate to a certain number of allele alterations, which is not surprising since the two methods rely on different approaches.

Our cgMLST analysis of a previously reported B. pseudomallei outbreak in Australia (7) demonstrates its potential to identify the source of an infection. However, in this example, B. pseudomallei SNP-indel-based analysis showed a slightly higher resolution than cgMLST (Fig. 3) since the latter could not resolve the environmental strains from the same property (7). Despite this potential slight difference in resolution, we demonstrate that cgMLST enables the effective molecular investigation of all the epidemiological settings reevaluated here depending on high-resolution typing.

We then applied the cgMLST scheme to investigate a recent melioidosis case in Vietnam (Fig. 4). cgMLST analysis of the clinical isolate and epidemiologically linked environmental strains revealed seven environmental isolates, which differed from the patient strain in just 3 to 5 alleles, pinpointing the sugarcane field of the patient as the source of infection. The two clinical isolates (NA76 [blood] and NA77 [pus]) from the same patient revealed no differences in the analyzed core genome genes. This observation is not surprising as polyclonal B. pseudomallei infections are considerably rare (4). The investigated patient had a melioidosis relapse several months after the initial infection. The isolated strain (NA18) shared an identical allelic profile with the initial clinical isolates (NA76 and NA77), indicating relapse rather than reinfection. Besides the discovery of the environmental strain linked to the infection, we found two additional environmental cgMLST profiles in the same sugarcane field, which differed from the clinical isolates in many thousands of alleles. This demonstrates a high genetic diversity among Vietnamese strains found within a small geographic region.

Investigating the global B. pseudomallei population by cgMLST revealed a population structure comparable to that found by SNP-based analyses (5). As cgMLST schemes are highly standardized and cgMLST trees are easily expandable, our cgMLST scheme provides the basis for expanding the currently available global phylogenetic tree and in turn advancing our understanding of the global B. pseudomallei transmission pattern and endemicity. This will build the foundation to create clinical awareness and induce diagnostic capacity building, as adequate and early treatment is essential for successful therapy.

Our cgMLST scheme was implemented in Ridom SeqSphere^+^, providing a standardized nomenclature and thereby the basis for highly concordant and comparable results. New alleles are assigned immediately after typing new genomes in Ridom SeqSphere^+^. The construction of phylogenetic trees is automated in Ridom SeqSphere^+^, and trees are easily expandable by new isolates without the need for reanalysis. The new cgMLST scheme has been made public at https://www.cgMLST.org. Furthermore, it will be indexed by Pathogenwatch (https://pathogen.watch) and thereby made accessible for analysis by any user.

In conclusion, we established a cgMLST-based method applicable to clinical and environmental strains, which discriminates isolates on a very fine scale and shows high concordance with SNP-indel-based analysis. The high degree of standardization, the capacity to analyze hundreds of genomes with moderate computational power, and the simple expansion of current data sets will facilitate global collaborations in the molecular surveillance of B. pseudomallei. This might also have an impact on the identification of genetic profiles that are linked to virulence or associated with certain clinical manifestations or different environmental niches.
