# Supplementary material for: Development and Validation of a Burkholderia pseudomallei Core Genome Multilocus Sequence Typing Scheme To Facilitate Molecular Surveillance
Source: J Clin Microbiol. 2021 Jul 19;59(8):e00093-21. doi: 10.1128/JCM.00093-21 (PMC8373231; doi:10.1128/JCM.00093-21)
Supplement: Supplemental file 1 — Fig. S1. Download JCM.00093-21-s0001.pdf, PDF file, 336 KB [file jcm.00093-21-s0001.pdf]

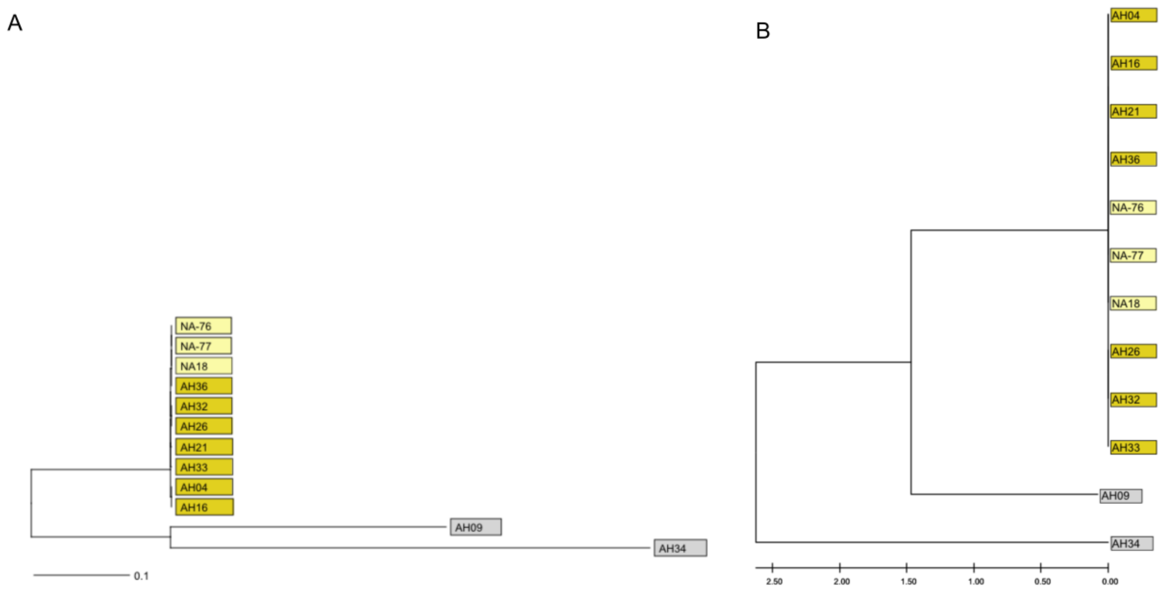

SI Figure 1: NJ Tree of a melioidosis outbreak in Vietnam based on cgMLST alleles (A) or core genome SNPs (B). Clinical isolates are shown in light yellow and environmental isolates in dark yellow or grey.
